# Immunoglobulin G responses against falciparum malaria specific antigens are higher in children with homozygous sickle cell trait than those with normal hemoglobin

**DOI:** 10.1186/s12865-019-0294-z

**Published:** 2019-04-27

**Authors:** George Msema Bwire, Mtebe Majigo, Robert Makalla, Lillian Nkinda, Akili Mawazo, Mucho Mizinduko, Julie Makani

**Affiliations:** 1 Department of Pharmaceutical Microbiology, School of Pharmacy, Muhimbili University of Health and Allied Sciences, Box 65001, Dar es Salaam, Tanzania; 2 Department of Microbiology and Immunology, School of Medicine, Muhimbili University of Health and Allied Sciences, Box 65001, Dar es Salaam, Tanzania; 3 Department of Hematology and Blood Transfusion, School of Medicine, Muhimbili University of Health and Allied Sciences, Box 65001, Dar es Salaam, Tanzania; 4 Ministry of Health, Community Development, Gender, Elderly and Children, Box 143, Babati, Manyara Tanzania; 5 Department of Epidemiology and Biostatistics, School of Public Health, Muhimbili University of Health and Allied Sciences, Box 65001, Dar es Salaam, Tanzania

**Keywords:** ELISA, Children, Dar Es Salaam, *Plasmodium falciparum*, Immunoglobulin G, Sickle cell

## Abstract

**Background:**

High Immunoglobulin G (IgG) response to *Plasmodium falciparum* antigens is associated with partial malaria protection in sickle hemoglobin (HbS) children. However, this response has been more studied in children with heterozygous sickle cell trait (HbAS) but little explored in those with homozygous sickle cell trait (HbSS). The current study was conducted to determine the IgG responses against specific *Plasmodium falciparum* antigens in children with homozygous sickle cell trait (HbSS) by comparing to those with normal hemoglobin (HbAA).

**Methods:**

A cross sectional study was conducted between April and July 2018 in Dar es Salaam tertiary hospitals. Parents were consented for their child to give about 5 ml of venous blood. IgG concentration from the blood plasma of 220 children (110 HbAA vs. 110 HbSS) were determined using indirect Enzyme Linked Immunosorbent Assay (ELISA). Then IgG medians were compared between the groups with prism 5 software (GraphPad) using Mann Whitney U test. Where the differences in age, hemoglobin levels and body weight between the groups was analyzed using independent sample t test. Multiple linear regressions were used to control cofounding variables such as body weight, age and hemoglobin level using statistical package for social sciences software (SPSS version 23). *P* value <0.05 was considered statistically significant.

**Results:**

The median IgG concentration to PfEBA-175, Pfg27, yPfs28C antigens were HbSS; 20.7 ng/ml (IQR; 18.1–25.6) vs. HbAA; 2.3 ng/ml (IQR; 1.21–3.04), HbSS; 2.76 ng/ml (IQR: 2.08–5.69) vs. HbAA; 1.36 ng/ml (IQR: 1.28–1.76), and HbSS; 26,592 ng/ml (IQR: 10817–41,462) vs. HbAA; 14,164 ng/ml (IQR; 3069–24,302) respectively (*p* < 0.0001 for all IgG). In both groups; age, body weight and hemoglobin level had no impact on the levels of IgG responses to *Plasmodium falciparum* antigens except for HbAA group which showed a significant increase in IgG against Pfg27 by 0.004 ng/ml with 1 g/dl increase in Hb level (*p* = 0.028).

**Conclusions:**

This study found significant higher levels of specific *Plasmodium falciparum* IgG responses in children with homozygous sickle cell trait than those with normal hemoglobin.

## Background

Malaria is caused by five species of Plasmodium*,* of the five species *Plasmodium falciparum* remains to be the major cause of childhood morbidity and mortality in sub-Saharan Africa [1]. In 2016, there were about 212 million malaria cases and 429,000 malaria deaths globally. Nearly 90% of all malaria cases and 92% of deaths occurred in Sub-Saharan Africa [2].

While the development of *Plasmodium falciparum* is partially inhibited in the sickle cell hemoglobin red cells, but still malaria is the commonest cause of hemolytic crises where less than 2% of the sickle cell disease children survive beyond 5 years of age with malaria morbidity ranging from 50 to 80% [3].

Each year, 300,000 children are born with sickle cell disease worldwide, 70% of them in sub-Saharan Africa [4]. Sickle cell, which is, inherited from parent(s) results to abnormality in the oxygen-carrying protein hemoglobin found in red blood cells [3, 4]. A child may inherit two abnormal copies of the haemoglobin gene (HbSS) or one abnormal haemoglobin gene (HbAS). Currently HbSS is a predominant genotype in sickle cell anemia (SCA) [4, 5].

Evidence from studies have reported the proportions of malaria in children with HbSS and HbAS are much lower when compared to children with normal hemoglobin (HbAA) [6, 7]. Innate factors such as restricted parasite invasion and/or growth in erythrocytes, enhanced phagocytosis of parasitized erythrocytes by macrophages, and impaired cytoadherence of parasitized erythrocytes to microvascular endothelial cells play roles for malaria protection especially in HbAS [8–10].

Moreover, recent findings suggest that malaria resistance in sickle hemoglobin (HbS) may also involve humoral immune responses [4, 6–8]. Higher levels of *Plasmodium falciparum* immunoglobulin G (IgG) responses have been shown to correlate with clinical protection in children with HbAS [9–12].

However, the role of IgG in HbSS is less characterized. Therefore, this study aimed at shedding more lights by determining the magnitude of malaria specific IgG responses in children with HbSS by comparing with HbAA genotype.

## Methods

### Study design and site

A cross-sectional study was conducted from April to July 2018 at Muhimbili National Hospital (MNH), Mwananyamala Referral Hospital, Temeke Referral Hospital and Mloganzila Academic Medical Center (MAMC) found in Dar es Salaam region. All these hospitals conduct a weekly sickle cell clinic in collaboration with Muhimbili University of Health and Allied Sciences (MUHAS) sickle cell programe.

### Study population

Children aged between 5 and 15 years tested malaria negative at the time of recruitment were enrolled to participate in this study. Consented parents/legal guardians of children with HbSS were requested to bring their siblings who are tested and known to have HbAA. In addition to that, children with hemoglobin levels below 6 g/dl, those treated for malaria infection a month prior to enrolment, and children participating in malaria vaccine trial were excluded from participating in this study.

### Sample size and sampling techniques

A total of 220 children were enrolled (110 HbSS and 110 HbAA) in this study. The multistage sampling was employed to first stratify hospital clinics (MNH, MAMC, Temeke and Mwananyamala) then participants were randomly selected from each clinic.

### Data collection

#### Hemoglobin level

Hemoglobin level was measured photometrically by using Hemocue Ltd., where children with hemoglobin concentration between 11.5 g/dl to 17.5 g/dl were designated as normal, 6 g/dl-11.4 g/dl were designated as mild anemic and below 6 g/dl were termed as severely anemic [13].

#### Malaria test

Screening for malaria was done by using rapid diagnostic tests (mRDT) (Access Bio, Inc., USA). Microscopy was performed for confirmation of malaria infection. For microscopy, thin and thick blood films were air-dried, Giemsa-stained, and examined by light microscopy (Thermo Fischer Scientific, USA) using 1000× oil- immersion lens for quantification and species identification. A participant who’s Hb counted above 6.0 g/dL and found malaria negative by microscopy was requested to give 5 ml of a venous blood for *Plasmodium falciparum* indirect ELISA.

### Determination of IgG responses to *Plasmodium falciparum* antigens

#### Plasma preparation from whole blood

A total of 5mls of whole blood were collected in an Ethylene-diamine-tetra-acetic acid (EDTA) tube (K3 EDTA, Labex, SRL, Italy), followed by centrifugation for 10 min at 1000–2000x g using a centrifuge. The resulted supernatant (plasma) was transferred into a clean polypropylene tube using a Pasteur pipette. Then samples were maintained at 2–8 °C while handled. Where plasma was not analyzed immediately was stored at − 80 °C or lower.

#### Enzyme linked immunosorbent assay (ELISA)

Indirect ELISA was performed as explained in a study by Akpogheneta et al [14]. Briefly, Nunc™ plates were coated overnight at 4 °C with 0.5 μg/ml of PfEBA-175, Pfg27 and yPfs28C antigens (BEI resources, USA) in protein buffered saline (PBS) (Thermo Fischer Scientific, Waltham, USA) (pH 7.4). After overnight incubation, the plates were washed five times with 250 μl PBS, and blocked by adding 250 μl PBS containing 1% fetal bovine serum (FBS) (Sigma, St Louis, USA) to each well and incubated overnight. The plates were then washed five times and 250 μl of plasma diluted in PBS at 1:1000 for antibodies to PfEBA-175, Pfg27 and yPfs28C were added and incubated overnight at 4 °C. The plates were again washed and 100 μl of horseradish peroxidase- conjugated rabbit antibodies against human antibodies (Bio-Techne Brands, USA) were added and incubated for 3 h at room temperature. Detection of the antibodies was done by adding 100 μl tetra-methylbenzidine (TMB) substrate (put in the dark area), and the reaction stopped after 15 min with 20 μl of 2 M H_2_SO4 (KPL, Gaithersburg, USA). Absorbance was measured as optical density (OD) at 450 nm (Tecan, San Jose, USA).

Standard curve was generated from a serial 5-fold dilution of purified human IgG starting at 1.0 ng/ml (Bio-Techne Brands, USA) and all samples were simultaneously assayed in duplicate.

#### Data management and analysis

Data from case report forms (CRFs) were recorded on Microsoft excel. In addition to that, data from the ELISA reader were exported directly into excel then to statistical package for social sciences version 23 (SPSS Software, Chicago Inc., USA) and Prism 5 software (GraphPad Software, USA) for coding and performing statistical analysis. IgG responses were compared between the different groups with Prism 5 software by using Mann Whitney U test. Where the differences in age, hemoglobin levels and body weight between the groups was analyzed using independent sample t test. Multiple linear regressions were used to control cofounding variables such as body weight, age and hemoglobin level using SPSS version 23. A two-tailed *p* value < 0.05 was considered statistically significant.

### Ethical consideration

Ethical approval was obtained from Muhimbili University of Health and Allied Sciences, Senate subcommittee for Research and Publications (MUHAS). A written informed consent was obtained from parents of all children for giving venous blood about 5 ml and test for IgG against specific *Plasmodium falciparum* antigens. Additionally, children above 5 years were assented before enrolling them in this study. Children tested malaria positive and found anemic their results were immediately communicated to their attending physician for further management.

## Results

### Characteristics of study participants

Of 220 children enrolled into the study 54% were males. The mean age (±SD) of the participants was 9 ± 3.2 years. Children with HbSS were significantly younger 9. ± 3.1 years than HbAA whose mean age was 10 ± 3.2 years, *p* = 0.031. The mean body weight (±SD) of the participants was 24.9 ± 10.2 kg. Underweight was more prevalent in children with HbSS 22.1 ± 7.3 kg than in their counterparts 27.6 ± 11.8 kg, *p* < 0.0001. The mean hemoglobin level (±SD) of the participants was 10.1 ± 2.6 g/dl. Anemia was predominantly in children with HbSS 8.2 ± 1.5 g/dl than children with HbAA 12.0 ± 1.9 g/dl, *p* < 0.0001 (Table 1).

**Table 1:** Characteristics of study participants

*Characteristics*		*HbSS n (%)*	*HbAA n (%)*
*Sex*	Male	55 (50%)	71 (64%)
	Female	55 (50%)	29 (46%)
*Age (years)*	5–10	84 (77.0%)	66 (62.0%)
	11–15	26 (23.0%)	44 (48.0%)
*Weight (Kg)*	11–20	38 (30.9%)	51 (49.0%)
	21–30	21 (19.1%)	44 (40.0%)
	31–40	32 (32.0%)	12 (10.9%)
	41–50	15 (13.6%)	3 (2.7%)
	51–60	4 (3.6%)	0 (0%)
*Hb level (g/dl)*	6–8.9	79 (71.6%)	4 (3.6%)
	9–11.9	27 (25.3%)	7 (6.4%)
	≥ 12	4 (3.0%)	88 (80%)

### Magnitude of IgG antibody responses to PfEBA-175

**Fig. 1:**
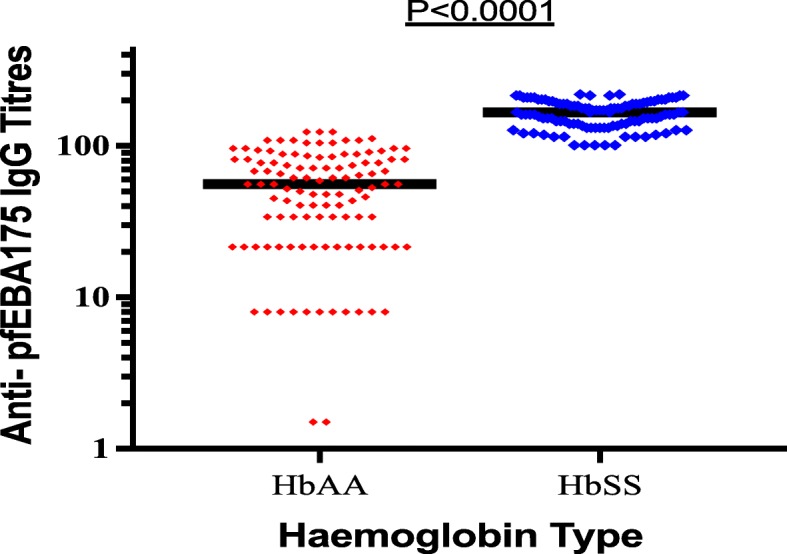
Median IgG levels against PfEBA-175 in health children with HbSS and HbAA living in malaria endemic region

The median levels of IgG specific responses to PfEBA-175 antigens were significantly higher in children with HbSS 20.7 ng/ml (IQR; 18.1–25.6) compared to children with HbAA 2.3 ng/ml (IQR; 1.21–3.04), *P* < 0.0001 (Fig. 1).

### Magnitude of IgG antibody responses to Pfg27

**Fig. 2:**
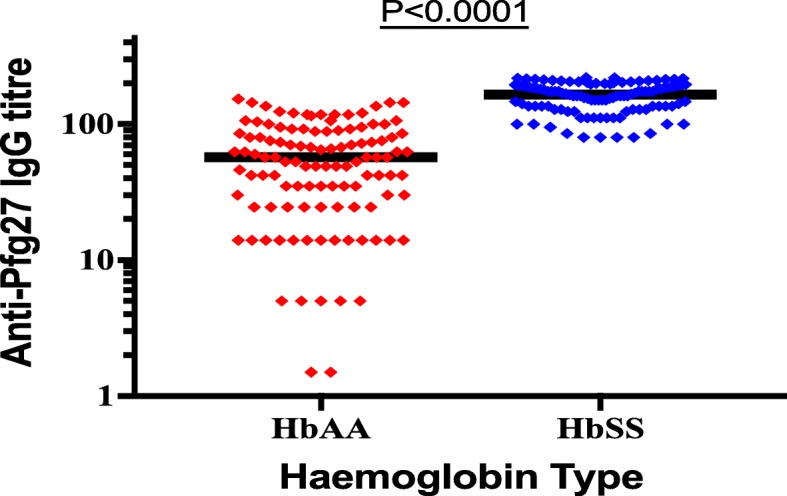
Median IgG levels against Pfg27 in HbSS and HbAA children living in malaria endemic region

The median levels of IgG responses to Pfg27 antigens were significantly higher in HbSS 2.76 ng/ml (IQR: 2.08–5.69) compared to HbAA 1.36 ng/ml (IQR: 1.28–1.76) group, *p* < 0.0001 (Fig. 2).

### Magnitude of IgG antibody responses to yPfs28C

**Fig. 3:**
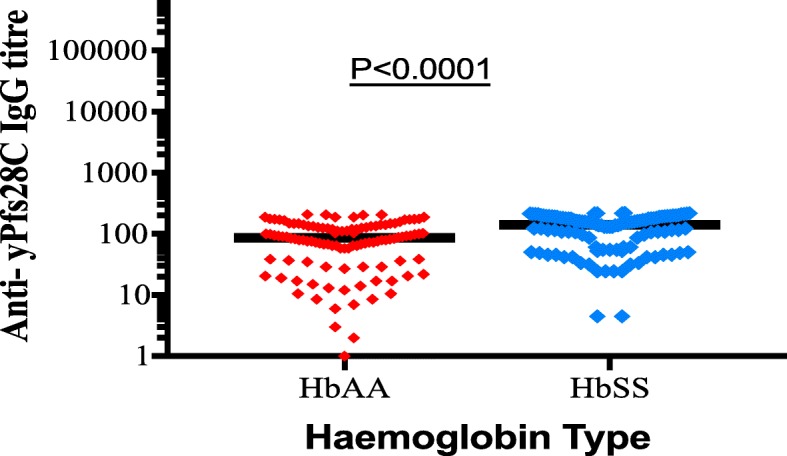
Median IgG levels against yPfs28C in health children with HbSS and HbAA living in malaria endemic region

Higher levels of IgG responses were observed in HbSS than in HbAA, the median levels.

of IgG responses to yPfs28C antigens were 26,592 ng/ml (IQR:10817, 41,462) compared.

to 14,164 ng/ml (IQR: 3069–24,302) in HbAA, *p* < 0.0001 (Fig. 3).

### Association between antimalarial IgG responses and age, body weight, and hemoglobin level

A multivariate regression model was used to control age, body weight and hemoglobin level to determine the relationship between hemoglobin genotype and IgG responses. Age, body weight, and hemoglobin levels did not significantly influence the magnitudes of IgG responses to PfEBA-175, Pfg27, and yPfs28C in children with HbSS. Similarly, age, body weight and hemoglobin level were found not to have a significant association with IgG response in children with HbAA (Tables 2, 3 and 4), except for HbAA children which showed a significant increase in IgG against Pfg27 by 0.004 ng/ml with an increase in Hb levels (*p* = 0.028) (Table 3).

**Table 2:** Table summarizing multiple linear regressions for IgG againist PfEBA-175. Increase in age, body weight and Hb level increased the concentration of IgG against PfEBA-175 by 0.501, 0.103 and 1.262 ng/ml respectively in children with HbSS but the increase was not statistically significant. On the other hand, there was a decrease in IgG concentration against PfEBA-175 by 0.0093 ng/ml with an increase in age but body weight and Hb level increased the IgG concentration by 0.01 and 0.046 ng/ml respectively although both decrease and increase were not statistically significant in children with HbAA

IgG against	Hemoglobin type	Factor	B	*P* - value
PfEBA-175 (ng/ml)	HbSS	Age (year)	0.501	0.582
		Body weight (kg)	0.103	0.282
		Hb level (g/dl)	1.262	1.086
	HbAA	Age (year)	−0.093	0.335
		Body weight (kg)	0.010	0.733
		Hb level (g/dl)	0.046	0.768

**Table 3:** Table summarizing multiple linear regressions for IgG againist Pfg27. Increase in age decreased the IgG concentration by 7.12 ng/ml but body weight and Hb level increased the concentration of IgG against Pfg27 by 3.118 and 2.223 ng/ml respectively in children with HbSS but both decrease and increase were not statistically significant. On the other hand, there was a decrease in IgG concentration against Pfg27 by 0.016 ng/ml with an increase in age but body weight and Hb level increased the IgG concentration by 0.006 ng/ml and 0.004 ng/ml (*p* = 0.028) respectively

IgG against	Hemoglobin type	Factor	B	*P* - value
PfG27 (ng/ml)	HbSS	Age (year)	−7.120	0.331
		Body weight (kg)	3.118	0.317
		Hb level (g/dl)	2.223	0.822
	HbAA	Age (year)	−0.016	0.118
		Body weight (kg)	0.006	0.07
		Hb level (g/dl)	0.004	0.028

**Table 4:** Table summarizing multiple linear regressions for IgG againist yPfs28C. Increase in body weight decreased the IgG concentration by 1040.428 ng/ml but age and Hb level increased the concentration of IgG against yPfs28 by 2499.702 and 3663.876 ng/ml respectively in children with HbSS but both decrease and increase were not statistically significant. On the other hand, there was a decrease in IgG concentration against yPfs28C by 110.651 ng/ml with an increase in Hb level but age and body weight increased the IgG concentration by 237.158 and 171.869 ng/ml respectively although both decrease and increase were not statistically significant in children with HbAA

IgG against	Hemoglobin type	Factor	B	*P* - value
**yPfs28C (ng/ml)**	HbSS	Age (year)	2499.702	0.385
		Body weight (kg)	−1040.428	0.395
		Hb level (g/dl)	3663.876	0.345
	HbAA	Age (year)	237.158	0.693
		Body weight (kg)	171.869	0.355
		Hb level (g/dl)	−110.651	0.909

## Discussion

To the best of our knowledge, this was the first study in our settings undertaken to determine the magnitude of IgG levels directed against PfEBA-175, Pfg27 and yPfs28C in children with homozygous sickle cell trait and those with normal hemoglobin.

The magnitude of malaria remains high and affects up to about 40% of the world’s population with around 300–500 million people currently infected and mainly in the tropics [4]. Several studies have mentioned the need to develop an effective human malaria vaccine for the control and possible eradication of malaria across the globe aiming at reducing the morbidity and mortality associated with the disease and protecting those at risk [22–24, 28]. Studies on the immunological responses to various malaria parasite antigens are needed and these should be inclusive of individuals with haemoglobinapathies [8, 16–19].

This study has therefore shaded more light in understanding how the natural malaria protection (IgG levels) can be affected by differences in hemoglobin shape. Blood stage antigens PfEBA-175, Pfg27 and yPfs28C, which are also candidates for malaria vaccine, were employed to explore the differences [25–29].

PfEBA-175, a blood stage malaria vaccine candidate and a parasite ligand at the apical end of the *Plasmodium falciparum* merozoites enhance the binding of parasites to the surface of a red blood cell during merozoites stage [16]. Antibodies directed against this antigen prevent further binding of merozoites to the red blood cell surface [20].

The current study found the median IgG antibody responses to PfEBA-175 were lower 2.29 ng/ml (IQR: 1.21–3.04) in HbAA than 20.68 ng/ml (IQR: 18.1–25.64) in HbSS. These findings correlate from those reported by Howaida et al. which found an increased mean IgG level of IgG 13.3 pg/ml in children with sickle hemoglobin versus 4.5 pg/ml normal children [8]. However another study conducted in Senegal found lower levels of IgG1 and IgG3 in individuals with sickle cell trait, limited exposure to malaria was reported to be associated with this findings [30]. In addition to that, findings from Senegal imply that different IgG subclasses may have different immune responses directed against malaria antigens however the current study was set to determine the total IgG.

Pfg27, a gametocyte-specific protein is expressed early during sexual differentiation, this protein plays a crucial role in the sexual development of *P. falciparum* and parasites lacking its gene fail to develop sexually [21]. This study found median IgG levels against Pfg27 was 1.36 ng/ml (IQR: 1.28–1.76) in HbAA and 2.76 ng/ml (IQR: 2.08–5.69) in HbSS. This means that children with HbSS have an enhanced immune response against gametocyte specific protein.

Pfs28 a sexual malaria vaccine candidate which has shown to block malaria transmission in gut by preventing ookinete development during sexual stage [15]. IgG against yPfs28C were found to be more enhanced in children with HbSS 4164 ng/ml (IQR: 3069–24,302) than 26,592 ng/ml (IQR; 10,817–41,462) in HbAA. Despite of having no literature tested these antigens Pfg27 and yPfs28C in similar fashion as reported by W. Usener form BEI Resources that donated these antigens (personal communication, April 05, 2018). But these findings correlate to other findings, which employed the use of other antigens and found higher IgG levels in sickle hemoglobin individuals [8, 30, 32].

This study also found no association between age, body weight and hemoglobin and increase or decrease in IgG response against PfEBA-175, Pfg27 and yPfs28C in both HbSS and HBAA children except for HbAA children showed a significant increase in IgG against Pfg27 by 0.004 ng/ml with an increase in Hb levels. These findings differ from the study, which found a significant association between an increased IgG levels versus weight and hemoglobin level. IgG levels in this study increased with an increase in Hb levels and weight. These indicated that other factors such as weight, Hb levels apart from sickle cell type may also influence the increase in IgG, however the current study only found an association between Hb level and IgG against Pfg27 in HbAA where there was an increase of 0.004 ng/ml with an increase in Hb levels, the discrepancy may have been contributed by other factors such as use of different antigens, geographical area and study population [30–33].

Furthermore, increased IgG levels in sickle cell individual have been associated with partial malaria protection; hence, treatment of individual with malaria, regardless of their sickle cell status, is recommended.

## Limitations

This study was limited by unavailability of serum from a person who has never been exposed to malaria to define a cutoff for positive and negative response to each antigen. To mitigate this Ig concentration from both HbSS and HbAA were explored from the standard curve and compared.

In addition to that, the conclusion of the findings from this study is limited to HbSS and HbAA children because HbAS were not included in this study.

## Conclusions

In conclusion, this study found that humoral response (IgG) against specific *Plasmodium falciparum* parasite antigens is more enhanced in children with HbSS than HbAA.

This study recommends further study to characterize IgG subclasses against PfEBA-175, Pfg27 and yPfs28C. In addition to that, this finding suggests further study to be conducted to rule out the influence of hemoglobin polymorphism in natural immunity against *Plasmodium falciparum* and implications for vaccine development.

## Abbreviations

BEI: Biodefense and Emerging Infections
HbAA: Normal hemoglobin
HbAS: Heterozygous sickle hemoglobin
HbSS: Homozygous sickle hemoglobin
IQR: Interquartile range
PfEBA-175: Recombinant *Plasmodium falciparum* erythrocyte binding antigen
Pfg27: Recombinant *Plasmodium falciparum* gametocyte protein
SD: Standard deviation
yPfs28C: Recombinant *Plasmodium falciparum* zygote and ookinete surface protein
